# Capturing ‘R&D excellence’: indicators, international statistics, and innovative universities

**DOI:** 10.1007/s11192-017-2602-9

**Published:** 2017-12-02

**Authors:** Robert J. W. Tijssen, Jos J. Winnink

**Affiliations:** 1 Center for Science and Technology Studies (CWTS), Leiden University, Leiden, The Netherlands; 2 DST-NRF Center of Excellence in Scientometrics and Science, Technology and Innovation Policy, Stellenbosch University, Stellenbosch, South Africa

**Keywords:** National science systems, Research universities, Science-technology linkages, Patents, Bibliometrics

## Abstract

Excellent research may contribute to successful science-based technological innovation. We define ‘R&D excellence’ in terms of scientific research that has contributed to the development of influential technologies, where ‘excellence’ refers to the top segment of a statistical distribution based on internationally comparative performance scores. Our measurements are derived from frequency counts of literature references (‘citations’) from patents to research publications during the last 15 years. The ‘D’ part in R&D is represented by the top 10% most highly cited ‘excellent’ patents worldwide. The ‘R’ part is captured by research articles in international scholarly journals that are cited by these patented technologies. After analyzing millions of citing patents and cited research publications, we find very large differences between countries worldwide in terms of the volume of domestic science contributing to those patented technologies. Where the USA produces the largest numbers of cited research publications (partly because of database biases), Switzerland and Israel outperform the US after correcting for the size of their national science systems. To tease out possible explanatory factors, which may significantly affect or determine these performance differentials, we first studied high-income nations and advanced economies. Here we find that the size of R&D expenditure correlates with the sheer size of cited publications, as does the degree of university research cooperation with domestic firms. When broadening our comparative framework to 70 countries (including many medium-income nations) while correcting for size of national science systems, the important explanatory factors become the availability of human resources and quality of science systems. Focusing on the latter factor, our in-depth analysis of 716 research-intensive universities worldwide reveals several universities with very high scores on our two R&D excellence indicators. Confirming the above macro-level findings, an in-depth study of 27 leading US universities identifies research expenditure size as a prime determinant. Our analytical model and quantitative indicators provides a supplementary perspective to input-oriented statistics based on R&D expenditures. The country-level findings are indicative of significant disparities between national R&D systems. Comparing the performance of individual universities, we observe large differences within national science systems. The top ranking ‘innovative’ research universities contribute significantly to the development of advanced science-based technologies.

## Introduction

### R&D excellence

High-level national policy debates and decisions on science and innovation policies often hinge on statistical information about a country’s R&D expenditure levels. R&D input/output models and performance statistics usually relate to domestic R&D systems or national economies and use system-level aggregate statistics as computational parameters. Acronyms like GERD, BERD and HERD tend to dominate policy reports,^1^ each of which are crude indicators of R&D performance and—by definition—uninformative about realized R&D outputs and socioeconomic benefits of research and development. Where most statistics and performance indicators fall into two main categories—either scientific research outputs (R) or technological development outputs (D)—this paper introduces ‘R&D excellence’ as a new measure that combines both domains. This metric captures the upper regions of a R&D performance distribution, the zone of technical ingenuity and cutting-edge know-how that contributes to the creation of highly innovative inventions and advanced technologies. We define ‘R&D excellence’ as: “the ability to produce scientific research that contributes to the development of innovative technologies”.

By definition, only a tiny share of the world’s scientific research effort is regarded as a genuine breakthrough discovery (often recognized with the benefit of several years of hindsight); the same is true for ‘game changing’, innovative technologies that tend to create highly valuable, long-lasting socioeconomic impacts. Although some studies have been done (e.g. Griliches 1990; Adams and Griliches 1996), there is no accepted econometric model, consolidated statistical database, or analytical tool available to assess R&D excellence within countries or economies. Given the difficulties in operationalization the underpinning general concept of ‘excellence’—which is fraught with conceptual, theoretical and methodological difficulties (Jackson and Rushton 1987; Tijssen 2003)^2^—very little quantitative, empirical studies have been done to unravel these peak levels of creative achievement (Lhuillery et al. 2016).

Measuring R&D excellence is methodologically challenging, especially within an internationally comparative framework. In this publication we introduce an analytical model and measurement method to help fill this information gap. Our R&D excellence model is based on two quantitative proxies of R&D outputs: (a) research publications in scientific and technical journals; (b) patents. Our macro-level empirical study is driven by a series of exploratory questions: can we identify structural factors to explain why some countries seem to excel in R&D excellence? Is the sheer size of the national R&D systems one of those success factors? Do the observed findings present general patterns and meaningful information, which lends itself for performance indicators that could be used for international benchmarking and R&D performance monitoring?

### Measurement model and information sources

We operationalize the concept ‘R&D excellence’ by extracting empirical information from knowledge flows between science and technology, more specifically from reference lists (‘citations’) in publications. The backward citations in patents are used to uncover the extent to which the patented inventions rely on scientific research publications in a patent’s referenced list of ‘non-patent literature’. Macro-level citation impact analyses requires large-scale bibliographic databases and of research publications, patents, and patent-to-publication references that connect these information items. An expanding body of academic work applies this source which has proved to be of great analytical power to measure general patterns and trends in systematic studies at macro-, meso- or micro-levels (e.g. Narin et al. 1997; Hicks et al. 2000; Tijssen et al. 2000; Cassiman et al. 2007; Van Looy et al. 2011; Ahmadpoor and Jones 2017). The different studies indicate the technology areas that are closest to science: pharmaceuticals and biotechnology, computer technology and digital communication, nanotechnology, and some fields of chemistry (OECD 2013).^3^

We quantify R&D excellence by focusing on upper percentiles of a statistical distribution in terms of frequency counts of literature references (‘citations’) between citing patents and cited research publications. Our citation data connect the world’s most highly cited patents (cited by other patents worldwide, across all technology areas) to publications in peer-reviewed scientific and technical journals. We focus on the top 10% most highly cited patents (denoted here as ‘TopTech patents’).^4^ Scientific research publications cited within those patents are denoted as ‘SciTopTech publications’. Many of these publications can be seen as qualifiable success stories of university research impact on technology development.

In our case, the citing patents were identified in the CWTS in-house version of PATSTAT database (all worldwide patents filed in 2004–2013, by earliest filing date within the family).^5^ The cited research publications, published in 2001–2013, were identified in Clarivate Analytics’ *Web of Science Core Collection* (WoS). It is important to note that high-quality ‘main stream’ research is sufficiently covered by the thousands of scientific journals that are indexed in the WoS.^6^ The citation counts are based on an integer counting scheme, where each cited publication is assigned in full to all countries mentioned in the author affiliation list of a publication.

## Empirical results

### Countries and national science systems

Based on our analysis of 4,351,180 patents and 13,742,865 research publications, we determined the quantities the SciTopTech publications per country. The left hand column of Table 1 presents a ranking of the countries^7^ that produce, relatively speaking, the largest numbers of SciTopTech publications.

**Table 1:** Top 20 countries by SciTopTech publication output. *Data sources*: Web of Science-Core Collection (WoS); PATSTAT (CWTS, Leiden University, Netherlands); information items: cited WoS-indexed publications (2001–2013); citing PATSTAT-indexed patent families in (2004–2013). Excludes research publications in the social sciences and humanities

	R&D excellence—absolute	R&D excellence—relative
	SciTopTech publication output count	% SciTopTech in total publication output
United States	225,721	3.6
Japan	56,662	4.0
Germany	51,277	3.3
United Kingdom	46,478	2.8
France	33,606	3.1
China	26,675	1.3
Canada	24,070	2.7
Italy	23,472	2.6
Netherlands	16,988	3.3
South Korea	16,128	2.7
Spain	15,529	2.2
Switzerland	14,448	3.8
Australia	13,541	2.2
Sweden	12,049	3.5
Belgium	9468	3.4
India	8667	1.4
Taiwan	7970	2.4
Israel	7654	3.8
Denmark	6998	3.4
Austria	6386	3.1

The USA is the largest contributor in absolute numbers, followed by Japan, Germany and United Kingdom. The US produces well over 200 thousand of those publications, accounting for 3.6% of its total WoS-indexed publication output during the years 2001–2013. This outcome is indicative of the overall strengths of the world’s largest science system in terms of its size of R&D resources, but it is less informative with regards to America’s relative performance.^8^ When normalizing the SciTopTech publications according to their share in the total publication output of the USA, we see several countries moving up in the right hand column: Switzerland, Israel, Denmark and Belgium. The science systems in these small countries are, relatively speaking, more productive than the US in generating SciTopTech publications.

The list of countries in Table 1 are the world’s leading ‘innovative’ nations. However, the statistical relationship between a country’s ranking in both columns is weak (Pearson correlation coefficient *r* = 0.21); apparently a size-corrected measure of R&D excellence paints a different picture, raising the question why some countries produce disproportionally large shares of SciTopTech publications. In this short paper we briefly address this question and examine some enabling factors that might explain these differences in R&D excellence.

### Explanatory factors of R&D excellence

The sheer volume of a country’s R&D system, and associated economies of size and scope, are two of the most obvious explanatory factors. The extent of the R&D linkages between the business sector and public research sector will also explain part of the differences between countries. The size and composition of national R&D expenditures is generally seen as relevant input indicator. High-quality country-level statistical data are scarce. We collected R&D expenditure data on each of the 20 countries in Table 1 from the OECD’s *Main Science and Technology Indicators* database. Additionally we computed statistics on an R&D linkage indicator: the number of ‘university-industry co-authored publications’ (UICPs), an output-based indicator of science-based cooperation (Tijssen 2012). These UICPs not only represent collaborative linkages and knowledge flows, but also the absorptive capacity within the business sector to utilize industry-relevant results of university research (including the knowledge and skills of contributing human resources through recruitment and job mobility). The UICPs that involve domestic firms reflect absorptive capacity within the national R&D system, where geographical proximity is seen as an important factor of collaboration and utilization (Arundel and Genua 2004).

The full set of explanatory variables includes:

*GDP per capita*: Gross Domestic Product per capita (2011);
*GERD*: Gross Expenditure on R&D (2011);
*BERD*: Business Expenditure on R&D (2011);
*HERD*: Higher Education Expenditure on R&D (2011);
*%HERD*–*firms*: Higher education R&D spending funded by business sector (2011);
*%UICPs*–*all firms*: Share UICs in the total scientific publication output (2013);
*%UICPs*–*domestic firms*: Share UICs in the total scientific publication output involving a domestic-based business enterprise as research partner (2013)

Table 2 presents the Pearson correlation coefficients between the variables. The size-dependent measure of R&D excellence (‘R&D excellence—absolute’) is strongly related to the size of R&D expenditures (especially GERD and BERD). When corrected for size, ‘R&D excellence—relative’ shows an expected positive relationship with (size corrected) GDP per capita. Based on these findings, one may conclude that R&D excellence is largely explained in terms of R&D investment volumes and the income level of a country. More surprising is the significant positive correlation with research cooperation between universities and domestically located business enterprises (‘%UICP—domestic firms’), which is probably also partially reflected in the positive correlation between ‘Research excellence—relative’ with ‘%HERD—firms’. However, the overall level university-industry research cooperation (with enterprises worldwide) is negatively correlated with ‘R&D excellence—relative’. Concluding, it seems that size-corrected R&D excellence is linked to the ability of country to produce an effective domestic R&D system with a large degree of research cooperation between local universities and industry.

**Table 2:** Pearson correlation coefficients (20 OECD countries). *Data sources*: Web of Science Core Collection (CWTS, Leiden University, Netherlands); Main Science and Technology Indicator (Organisation of Economic Cooperation and Development (OECD), Paris)

	1	2	3	4	5	6	7	8
1. R&D excellence—absolute								
2. R&D excellence—relative	0.21							
3. GDP per capita	0.04	**0.59**						
4. GERD	**0.88**	− 0.10	− 0.23					
5. BERD	**0.86**	− 0.06	− 0.26	**0.99**				
6. HERD	0.25	0.22	− 0.06	0.26	0.27			
7. %HERD—firms	0.44	0.32	0.14	0.36	0.36	**0.90**		
8. %UICP—all firms	− 0.09	− 0.53	− 0.50	0.32	0.35	− 0.02	− 0.21	
9. %UICP—domestic firms	− 0.04	**0.67**	0.48	− 0.20	− 0.20	0.25	0.23	− 0.36

Boldfaced figures: correlation coefficients significant at the 0.01 level (2-tailed); the selected countries are listed in Table 1

The question remains to what degree R&D excellence depends on the quality of the domestic science system rather than R&D expenditure levels. This information is not captured in OECD databases. Collecting data on a wider range of countries, now including middle-income countries, we replaced our OECD data by information on ‘science systems’ that was extracted from the 2010 annual *Executive Opinion Survey* (EOS) which was published by the World Economic Forum’s *Global Competitiveness Index 2011*–*2012* (GCI).^9^ The country-level performance indicators, each defined by scores on a Likert scale from 1 (‘very low’) to 7 (‘very high’), are:

*Survey*—*UIC*: (EOS item: ‘University–industry collaboration in R&D’);
*Survey*—*R&D human resources*: (EOS item: ‘Availability of scientists and engineers’);
*Survey*—*science system quality*: (EOS item: ‘Quality of scientific research institutions’)

Our sample involves 70 countries^10^ covered by the selected GCI survey data. The results of the correlation analysis are presented in Table 3. In terms of ‘Excellence—absolute’, both ‘%UICPs—domestic firms’ and ‘science system quality’ are important factors, both referring to the attractiveness of research institutes and universities as knowledge suppliers. However, after correcting for the size of country’s science base, ‘R&D excellence—relative’ scores correlate strongly with the survey-based ‘science systems’ variables. Interestingly, the strong positive correlation with ‘%UICPs—domestic firms’ has now almost disappeared, indicated that such research links with domestic industry are more likely to occur with the high-income economies. The significant correlation of ‘Survey—UIC’ suggests that university-industry cooperation, in a more general sense as perceived by corporate executives, is nonetheless related to R&D excellence levels within a country. The low correlation coefficient between ‘Survey—UIC’ and ‘%UICPs—domestic firms’ is a counterintuitive result which could be explained by differences in perspective (i.e. subjective views vs. empirical data).

**Table 3:** Pearson correlation coefficients (70 countries). *Data sources*: Web of Science-Core Collection (WoS); PATSTAT (CWTS, Leiden University, Netherlands); Global Competitiveness Index 2011–2012 (Executive Opinion Survey, World Economic Forum)

	1	2	3	4	5	6	7	8
1. R&D excellence—absolute								
2. R&D excellence—relative	0.25							
3. GDP per capita	0.18	**0.75**						
4. UIC output—all firms	0.10	**0.46**	0.28					
5. %UICPs—all firms	− 0.13	**0.72**	**0.61**	**0.47**				
6. %UICPs—domestic firms	− 0.10	**0.35**	0.15	**0.60**	**0.49**			
7. Survey—UIC	0.30	**0.79**	**0.73**	**0.35**	**0.63**	0.20		
8. Survey—R&D human resources	0.16	**0.68**	**0.53**	0.29	**0.50**	0.35	**0.59**	
9. Survey—Science system quality	**0.32**	**0.81**	**0.70**	0.34	**0.65**	0.23	**0.93**	**0.61**

Boldfaced figures: correlation coefficients significant at the 0.01 level (2-tailed)

The size-independent variable ‘R&D excellence—relative’ is clearly the superior indicator to compare countries. After correcting for inter-correlations between the various variables, via a linear regression analysis (see Table 4), three generic ‘key’ factors emerge: economic development level, university-industry connections, and science system quality. The latter of these three, being opinion-based and weakly operationalized, requires a further breakdown into its major constituent parts to identify its core determinants, which may pertain to the system’s overall level of research quality, research specialization profiles of a country’s ‘R&D excellent’ universities, breadth and depth of the entire research system (universities and non-university organizations), or one of many other organizational features. The next subsection takes a closer look at that institutional level.

**Table 4:** Linear regression model to explain R&D excellence (70 countries). *Data sources*: Web of Science-Core Collection (WoS); PATSTAT (CWTS, Leiden University, Netherlands); Global Competitiveness Index 2011–2012 (Executive Opinion Survey, World Economic Forum)

Explanatory variable	Standardized beta coefficient	Significance
GDP per capita	0.34	0.00
UICP output—all firms	0.10	0.06
%UICPs—all firms	0.24	0.01
%UICPs—domestic firms	− 0.17	0.85
Survey—UIC	− 0.56	0.25
Survey—R&D human resources	− 0.015	0.95
Survey—science system quality	0.97	0.04
Model fit: adjusted *R* ^2^	0.92	

### Science system quality and innovative universities

**Fig. 1:**
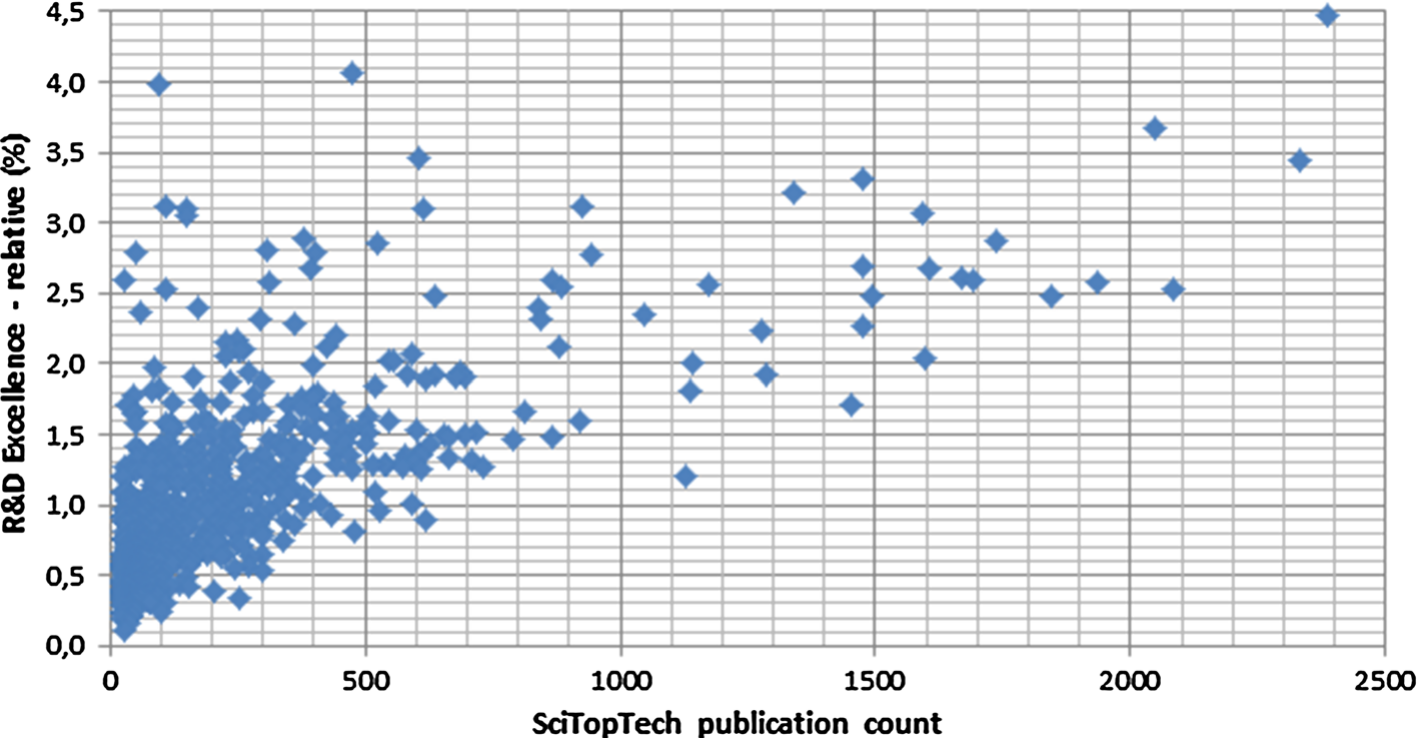
Relationship between ‘R&D excellence—relative’ and number of SciTopTech publications (716 universities worldwide). We removed two outliers: Harvard University (USA) which produced 6776 SciTopTech publications, and Rockefeller University (USA) with a 7.4% score on ‘R&D Excellence—relative’ (see Table 5)

Focusing on the highly significant contributions on ‘Survey—science system quality’ and UICP-based indicators, a nation’s university research system appears to be a prime explanatory factor of R&D excellence scores. One may expect large differences between universities, depending on their research specialization profile and linkages to industrial R&D.^11^ We refer to those universities that produce large numbers of SciTopTech publications as ‘innovative’. Figure 1 presents an overview of these numbers in relation to the ‘R&D Excellence—relative’ score. The selected set of 716 universities were extracted from the 2016 versions of the *Leiden Ranking* (www.leidenranking.com) and *U*-*Multirank* (www.umultirank.org) and comprise all universities with more than 25 SciTopTech publications in the years 2001–2013.

We find a very significantly positive relationship between both variables (*R* ^2^ = 0.48). The share of SciTopTech publications tends to be very low within a university’s total publication output (world group average = 1.0%). Several of the smaller research universities, with < 500 of SciTopTech publications, tend to be among the most innovative in relative sense.

Taking into account these general findings, one needs to correct for size of universities (‘large’ vs. ‘smaller’) and the nationality of universities (US vs. others) to present a more fair ‘like with like’ overview of innovative universities. Owing to differences in patenting practices and the numbers of SciTopTech publications, we distinguish the USA from other countries worldwide. As indicated above (see footnote 7), R&D excellence scores tend to over-represent those research publications that are cited in (US-filed) science-based patented technologies. These patents are concentrated in the biotechnology and pharmaceutical industry. Universities with high scores on R&D excellence therefore tend to be those with a strong focus the medical and life sciences research related to those industries and technology areas, which explains the top positions of Harvard University and Rockefeller University.

The top panel in Table 5 presents the top 5 large, research-intensive universities in the USA according to either their ‘R&D excellence—absolute’ or the ‘R&D excellence—relative’ score. The bottom panel lists the top 5 universities outside the US. Supplementing these well-known ‘R&D powerhouse’ universities in USA, we also find smaller European universities such as the University of Dundee in the United Kingdom, with only 306 SciTopTech publications in 2001–2013. While most prolific ‘innovative’ US universities produce three- or four-fold more SciTopTech publications than world average, universities in Europe and elsewhere produce at least twice as many. The relative underperformance of universities on the European continent may be also be partially due to the existence of large public research institutes (OECD 2011), such as the Fraunhofer Institutes in Germany, that are heavily engaged in industry-relevant R&D.

**Table 5:** Top 5 lists of R&D excellent ‘innovative’ universities: USA versus other countries. Data sources: Web of Science-Core Collection (WoS); PATSTAT (CWTS, Leiden University, Netherlands); Top 10% citing patents (2004–2013), cited research publications (2001–2013)

R&D excellence—absolute	SciTopTech output count	R&D excellence—relative	%SciTopTech output
Harvard University	6776	Rockefeller University	7.4
Massachusetts Institute Technology	2388	Harvard University	4.5
Stanford University	2331	Massachusetts Institute Technology	4.5
Johns Hopkins University	2084	University of Massachusetts Medical School	4.1
University of California, San Francisco	2047	University of California, San Francisco	3.7
University of Oxford (UK)	1690	University of Dundee (UK)	2.8
University of Cambridge (UK)	1474	University of Lausanne (CH)	2.8
University of Toronto (CA)	1452	Weizmann Institute of Science (IS)	2.7
University College London (UK)	1286	University of Oxford (UK)	2.6
Imperial College London (UK)	1136	London School of Hygiene & Tropical Medicine (UK)	2.3

Lower thresholds for inclusion: output of 5000 publications in 2001–2013, of which at least 50 SciTopTech publications. Excludes publications in the social sciences and humanities

Different rankings of universities emerge depending on the indicator. Correcting by the research output size changes the composition of the ranking, albeit only slightly in the case of the USA. Returning to the issue of explanatory factors, we assume that the industry-relevant R&D expenditure is a major determinant of R&D excellence—both absolute and relative. To explore this, and taking advantage of AUTM STATT database (AUTM 2017),^12^ on the research expenditure breakdown of major US universities we calculated correlation coefficients for the 27 US universities with more than 300 SciTopTech publications in 2001–2013 (see “Annex in Table 7”). The 2013 AUTM data relate to ‘Total Research Expenditure’, ‘Industrial Research Expenditure’, and ‘Gross License Income’. We assume that these 2013 expenditure levels are reasonably indicative for earlier years, but given the different time-periods between both types of data (and the lack of a time-lag in the analysis) the findings are merely a first indication of possible relationships. Given their relatively high explanatory value (see Table 4) we added the variables ‘UICP output—all firms’ and ‘%UICPs—all firms’ with data relating to 2009–2012.

Table 6 presents the Pearson correlation coefficients between the variables. ‘Total Research Expenditure’ has a relatively high (but non-significant) positive correlation coefficient (*R* = 0.38) with ‘R&D excellence—absolute’. This result is in line with Table 2 where the variable ‘HERD’ (Higher Education R&D expenditure) has a similarly modest positive correlation coefficient with ‘R&D excellence—absolute’. Generalizing from these exploratory results, there is no discernible indication that R&D excellence at US universities is heavily determined by any these variables, with the possible exception of total expenditure on research.

**Table 6:** Pearson correlation coefficients (27 universities in the USA). *Data sources*: Web of Science-Core Collection (WoS); PATSTAT (CWTS, Leiden University, Netherlands); AUTM (2017)

	1	2	3	4	5	6
1. R&D excellence—absolute						
2. R&D excellence—relative	**0.44**					
3. Total research expenditure	0.38	0.01				
4. Industrial research expenditure	0.07	− 0.03	**0.56**			
5. Gross license income	0.04	0.14	0.14	0.19		
6. UICP output—all firms	− 0.05	0.03	− 0.11	− 0.17	0.25	
7. %UICPs—all firms	0.23	0.16	0.06	0.04	0.27	0.37

Boldfaced figures: correlation coefficients significant at the 0.05 level (2-tailed)

## Concluding remarks

The empirical findings of this exploratory study reveal interesting general patterns across countries worldwide, notably the significant differences between high-income countries and middle-income countries. We also find large differences between universities. In both cases, a similar set of ‘structural’ factors seem to play a key role, but many questions remain as to the driving forces of R&D excellence. In this study we applied a simple descriptive model, where we assume that (1) the level of R&D excellence is determined by a very small set of explanatory factors, (2) all cited patents were analyzed collectively (irrespective of the patent system) and (3) no distinction is made between fields of science.

Hence, the results of our statistical analysis comes with two cautionary notes. First, R&D excellence scores, and rankings of ‘innovative’ universities, need to take into account (a) differences between research fields (where the medical and life sciences should be analyzed separately); (b) differences in how research publications are cited in patenting systems (where the USPTO patents should be analyzed separately). Secondly, the observed relational patterns between those factors between do not necessarily imply causality (in either direction), while the strength of these correlations are likely to be time-dependent, country-specific and sector-specific. Our crude explanatory model of R&D excellence may comprise of ‘enablers’, ‘catalysts’, ‘drivers’ and ‘accelerators’, each with a different role and impact on how science may contribute to technological development. Our study has not attempted to distinguish these various types, nor how they are likely to influence each other and create complex interdependent systems.

Acknowledging the study’s limited potential for generating transferrable lessons, and taking the above considerations into account, the main results do indicate that there are significant differences between high-income countries and low-income countries. Both size-dependent and size-independent performance measures are needed for a more comprehensive and balanced view of R&D excellence at this macro-level, where the size-corrected ‘R&D excellence—relative’ score is much higher correlated with science-related factors. Although our small set of explanatory factors offers some relevant insights as to why countries seem to excel, a more sophisticated model is required to help explain these empirical findings. Such a ‘systems characteristics’ view will require extensive macro-level econometric studies of a highly complex and dynamic global R&D system, including spill-over effects from other countries and interdependencies with business sector innovation systems.

The fundamental issue, and deeper analytical question, that emerges from this study is: what does our particular definition of R&D excellence actually represent, and how valid are our metrics and quantitative indicators? In the absence of any causal, detailed information on the actual driving forces or essential framework conditions, it remains unclear to what degree our explanatory factors are in fact crucial to create or enhance R&D excellence—either at the level of countries or within universities. Comparative measurement and large-scale benchmarking can only go so far; in-depth understanding requires information from supporting case studies. Ultimately we need ‘narratives with numbers’ to contextualize and interpret country-level statistics and to determine which factors are vital to R&D excellence within research environments and research-intensive organizations (Schmidt and Graversen 2017).

## Annex

See Table 7.

**Table 7:** Selected US universities and their R&D excellence statistics

	SciTopTech output count (2001–2013)	%SciTopTech output (2001–2013)
Harvard University	6776	4.5
Massachusetts Institute of Technology	2388	4.5
Stanford University	2331	3.4
Johns Hopkins University	2084	2.5
Michigan State University	1599	2.0
Duke University	1477	2.7
Cornell University	1276	2.2
Northwestern University	1046	2.3
Baylor College of Medicine	926	3.1
New York University	846	2.3
Emory University	842	2.4
California Institute of Technology	677	1.9
Ohio State University	663	1.3
Rutgers State University—New Brunswick	630	1.4
Rockefeller University	619	7.4
Icahn School of Medicine—Mount Sinai	613	3.1
Pennsylvania State University	612	1.3
Case Western Reserve University	592	2.1
Princeton University	546	2.0
Arizona State University	473	1.3
Iowa State University	457	1.5
Tufts University	443	2.2
Oregon Health and Science University	423	2.1
Brown University	400	1.8
Georgia Institute of Technology	360	1.3
Stony Brook University—SUNY	349	1.7
Texas A&M University—College Station	344	0.9

## Footnotes

1 GERD = Gross Expenditure on Research and Development; BERD = Business Expenditure on Research and Development ; HERD = Higher Education Expenditure on Research and Development.

2 For the sake of simplicity we will use the Wikipedia definition of ‘excellence’: “a talent or quality which is unusually good, and so surpasses ordinary standards” (Wikipedia website accessed on 11 October 2016).

3 Where ‘closeness’ is measured as the share of references in patents to scientific research publications of a among all the patent’s references to other documents.

4 Our choice for the top 10% percentile is somewhat arbitrary. The main practical consideration was to set the threshold at a level that produced a sufficiently large number of SciTopTech publications for internationally comparative studies, at the level of countries and research universities.

5 The citing patents are delineated in terms of ‘patent families’, where similar patents filed at different patent offices are aggregated into a single, de-duplicated entity. The PATSTAT database comprises several national and international databases, including USPTO (USA), EPO (Europe) and WIPO (worldwide).

6 We excluded research publications from research fields in the social sciences, arts and humanities, as they are rarely cited in patents but may nonetheless contribute significantly to a country’s publication output. Removing these publications from the output total avoids the bias of under representing the degree of R&D Excellence in countries with a high share of publications in these fields.

7 The country is assigned to a publication according to the address of the organizational affiliation of the authors. Each publication is assigned in full to all countries that are mentioned in author addresses.

8 The no 1 position of the USA is partially a result of the fact that many US universities file their patents at the US Patent Office (USPTO) that requires a larger numbers of references to relevant (US) research publications, often provided by the patent inventors. Other international patent systems, such as EPO in Europe, require much smaller numbers, where such inputs are provided by patent attorneys and/or inventors.

9 Given the inevitable degree of subjectivity of these executive views and opinions, one needs to be cautious with regards to the representativeness of this information source, and hence the robustness of our analytical findings.

10 The criterion for selecting countries: at least 100 research publications from 2001 to 2013, where the country is mentioned as an author affiliate addresses, were referenced in top 10% most highly cited patents.

11 A precursor study by Tijssen et al. (2016) compared and ranked research-intensive universities worldwide according to a series of ‘University—industry R&D linkage’ metrics. One of those measures, labelled by its technical acronym ‘%NPLR–HICI’, is identical to the ‘R&D excellence—relative’ metric in this article.

12 Similar type research expenditure data does not exist for a systemic comparison across a large range of non-US universities.
